# A Terrestrial Single Chamber Microbial Fuel Cell-based Biosensor for Biochemical Oxygen Demand of Synthetic Rice Washed Wastewater

**DOI:** 10.3390/s16010101

**Published:** 2016-01-15

**Authors:** Washington Logroño, Alex Guambo, Mario Pérez, Abudukeremu Kadier, Celso Recalde

**Affiliations:** 1 Centro de Investigación de Energías Alternativas y Ambiente, Facultad de Ciencias, Escuela Superior Politécnica de Chimborazo (ESPOCH), Panamericana Sur Km 1 ½, Chimborazo EC060155, Ecuador; alexfernag@gmail.com (A.G.); map_1090@hotmail.com (M.P.); crecalde672000@yahoo.com (C.R.); 2 Faculty of Science and Informatics, University of Szeged, Közép fasor 52, Szeged H-6726, Hungary; 3 Department of Chemical and Process Engineering, Faculty of Engineering & Built Environment, National University of Malaysia (UKM), UKM Bangi, Selangor 43600, Malaysia; abudoukeremu@163.com; 4 Escuela de Ingeniería Ambiental, Universidad Nacional de Chimborazo, Chimborazo EC060103, Ecuador

**Keywords:** microbial fuel cell, biological oxygen demand (BOD), biosensor

## Abstract

Microbial fuel cells represent an innovative technology which allow simultaneous waste treatment, electricity production, and environmental monitoring. This study provides a preliminary investigation of the use of terrestrial Single chamber Microbial Fuel Cells (SMFCs) as biosensors. Three cells were created using Andean soil, each one for monitoring a BOD concentration of synthetic washed rice wastewater (SRWW) of 10, 100, and 200 mg/L for SMFC1, SMFC2 and SMFC3, respectively. The results showed transient, exponential, and steady stages in the SMFCs. The maximum open circuit voltage (OCV) peaks were reached during the elapsed time of the transient stages, according to the tested BOD concentrations. A good linearity between OCV and time was observed in the increasing stage. The average OCV in this stage increased independently of the tested concentrations. SMFC1 required less time than SMFC2 to reach the steady stage, suggesting the BOD concentration is an influencing factor in SMFCs, and SMFC3 did not reach it. The OCV ratios were between 40.6–58.8 mV and 18.2–32.9 mV for SMFC1 and SMFC2. The reproducibility of the SMFCs was observed in four and three cycles for SMFC1 and SMFC2, respectively. The presented SMFCs had a good response and reproducibility as biosensor devices, and could be an alternative for environmental monitoring.

## 1. Introduction

Microbial fuel cells (MFCs) are an innovative technology due to their versatile and promising environmental applications such as bioremediation [1], as biosensors [2,3], for wastewater treatment [4,5] and for supplying energy for small devices [3,4]. The MFC is a bio-electrochemical device that converts organic substrates into electricity through the metabolism of electrochemically active microorganisms [5,6]. The process into an MFC is as follows: (a) the organic compounds are oxidized in the anode chamber of the MFC; (b) electrons produced by microorganisms are transferred to the anode electrode and subsequently to the cathode electrode through an external circuit; (c) protons are diffused across the proton exchange membrane (PEM) from the anode to the cathode chamber; and (d) protons and electrons react with atmospheric oxygen for the electrochemical balance in the cathode chamber by forming H_2_O. MFCs can be configured as a double or single chamber device, and can be inoculated with sediment, soil or wastewater. Pure and mixed cultures have been tested in the MFC technology, which may metabolize a small and wide range of substrates, respectively.

Biological oxygen demand (BOD) is a well-known indicator of water quality. The BOD refers to the amount of oxygen needed by microorganisms to oxidize the organic matter present, and conventionally is analyzed by the standard methods of the U.S. Environmental Protection Agency [7]; however, it is a time consuming method [8].

MFC-based biosensors have been tested for monitoring volatile fatty acids [9], COD [10], chromium, iron, nitrate, sodium acetate [11], toxic components [12], anaerobic digestion processes [13], biodegradable organic matter [14], BOD [15], water quality cadmium [16] and Cu (II) [17]. At the same time, MFC-based biosensors have limitations for commercial application due to the unavailability of standardized bacterial mixtures, although a known bacterial mixture has been used for measuring BOD in wastewater showing a good accuracy and reproducibility [8]. Although the lower current output of MFCs is recognized as a limiting factor for their application, an energy management system for amplifying the voltage up to 5 V was reported [18]. Addionally, mixed cultures from wetland soils have also been used to degrade complex organic substrates considering their higher metabolic activity, where the output voltages were acceptable according to the reported in databases [19,20].

This study tested if soil microorganisms from the Andean wetlands can respond in terrestrial single chamber microbial fuel cells (SMFCs)-based biosensors to BOD concentrations of SRWW, through the bioelectricity generation performance. Data were collected over a testing time of 15 d, looking for the feasibility for reaching the early steady stage of this kind of MFC as a biosensor.

## 2. Experimental Section

### 2.1. Experimental Setup

Andean soil has previously been used for inoculating MFCs to generate bioenergy by using organic solid wastes as a microbial fuel. The microbial inoculum was determined to be a mixed consortium, sampled from undisturbed Andean soils at 3850 m above sea level (masl); 763138W–9833826N, and collected between 20 to 40 cm depth [20]. The soil used in this study presented the following characteristics: pH 5.4, organic matter 2.0%, electrical conductivity 170 μS, ammonia nitrogen 9.1 mg/L, phosphorous 40.3 g/L and potassium 0.96 mg/L. A contrast of physico-chemical properties was done between the present and previous studies, and most of the parameters were within the same range. Colony forming units (CFU/g soil) were determined according to Logroño *et al.* [20]. Although, the same soil was used for assembling all SMFCs, the initial CFU/g soil counts were determined to be 3.3 × 10^9^, 5.0× 10^9^ and 5.0× 10^8^ CFU/g soil for SMFC1, SMFC2 and SMFC3, respectively. The microbial population and diversity of the SMFCs were supposed to be different. This factor limited any further comparison between OCV and BOD concentration tested in the cells.

### 2.2. MFC Assembly and Monitoring

**Figure 1:**
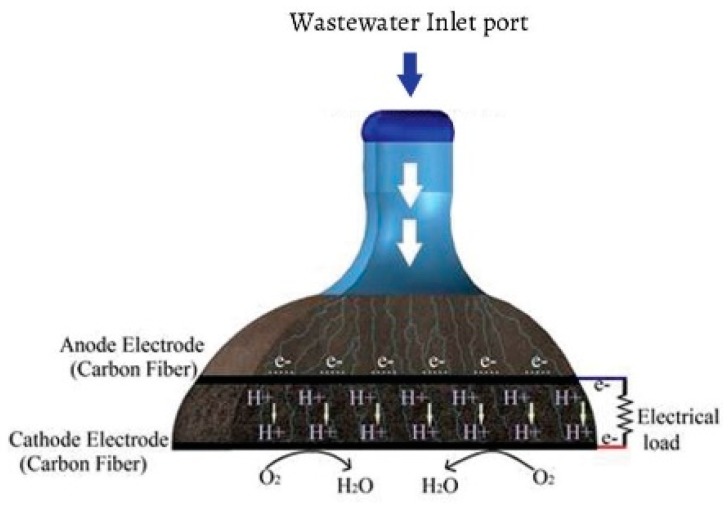
Schematic of the SMFCs installed for BOD monitoring.

Three independent SMFCs were simultaneously installed at the ESPOCH Biotechnology Lab, where the temperature fluctuated between 25 to 28 °C and altitude was 2700 masl. The cells were made of polyethylene containers of 3900 cm^3^ volume. Figure 1 depicts a scheme of the SMFCs tested. The inlet port was installed in the anode chamber for wastewater feeding. The anode chamber was filled with 2.1 kg of soil, where the soil microorganisms metabolized the wastewater, producing electrons and protons. The SMFCs employed a blend of soil-activated carbon as membrane, 2.1 kg and 120 g, respectively. Both, anode and cathode electrodes were made of carbon fiber (300 mm × 250 mm), keeping them equal in the SMFCs, and the distance among them was 50 mm. The anode and cathode were connected with two iron wires forming an external circuit. The OCV was measured by using a DT-832 digital multimeter [18,19]. The oxidant (O_2_) in the cathode was supplied from the atmosphere during the elapsed time of the experiment. Data were collected for 90 min, every 5 min after inoculation of SRWW for over a testing time of 15 d (considering each day as an independent cycle).

### 2.3. Wastewater Preparation and Inoculation

Three BOD concentrations of SRWW were prepared by washing two types of commercial rice (A, B) with mineral water (MW; Tesalia Springs Company, Riobamba, Ecuador). The SRWW was analyzed as shown in Table 1. The composition of MW was as follows: Ca^2+^ 22 mg/L, Mg^2+^ 58 mg/L, Na^+^ 51 mg/L, P^+^ 5.2 mg/L, HCO_3_^−^ 380 mg/L, SO_4_^2−^ 27 mg/L, Cl^−^ 32 mg/L, DS 440 mg/L. MW alone was supplied in the control SMFC.

The commercial rice B was better than A for reaching constant BOD concentrations of 10, 100 and 200 ppm during the experimentation. Each SMFC tested one independent concentration. Due to the fact the pH and BOD might change during the time; the SRWW supplied into the SMFCs was renewed in each independent cycle during the elapsed time of 15 d. Effects of different pH of SRWW were not studied in the presented article.

The feed rate should be well below the fuel saturation conditions, because a high feed rate causes incomplete fuel consumption. On the other hand, the oxidant supply can limit the cathode reaction [21]. Thereby, in this study the Andean soil inside the SMFCs was saturated with SRWW at a feed rate of 94 mL every 24 h in order to avoid overflowing or supersaturation of the SMFC matrix. The SRWW descended by gravity from the anode to the cathode.

**Table 1:** Biochemical Oxygen Demand concentrations in the SRWW.

SMFC	Mixing Time (min)	Concentration (ppm)	Analytical Method
SMFC1	5	10 (1000 mL of MW + 14.175 g rice)	Standard Method PEE/LS/CF/01
SMFC2	5	100 (1000 mL of MW + 56.7 g rice)	Standard Method PEE/LS/CF/01
SMFC3	5	200 (1000 mL of MW + 226.8 g rice)	Standard Method PEE/LS/CF/01

## 3. Results and Discussion

Electricity generation and reproducibility was achieved in SMFCs while SRWW was inoculated as carbon source (the effect of different wastewaters was not studied). During the elapsed time of 15 d (15 cycles) three major stages of OCV output were observed in the SMFCs: transient, linearly increasing and steady; then we related it with the microbial growth curve, latency, exponential and stationary, respectively. Good linearity between current and BOD in MFCs inoculated with concentrations higher than 100 mg/L, as reported by Chang *et al.* [15] was not been observed. Likewise, in this study only SMFC1 (10 ppm) and SMFC2 (100 ppm) reached the steady stage, each one on different days; suggesting that BOD concentration could be an influencing factor. Starch was considered to be the main compound and the electron donor of the SRWW in the SMFCs, therefore Equation (1) could illustrate the reactions involved in the SMFCs. Due to the fact the SMFCs in this study were inoculated with soil, the OCV could be generated by extracellular electron transfer [22], and such mechanisms have been reported for soil bacteria belonging to *Proteobacteria* and *Firmicutes* phylum [23]:

(1) $$C_{6}H_{10}O_{5}+7H_{2}O\to 6CO_{2}+24H^{+}+24e^{-}$$

OCV showed a highly variable transient trend named Phase 1. The period of the transient stage was the same for all SMFCs (7 d), but each one behaved independently, likely due to the fact the effectbes consume the only carbon source in the soil. ed and lacking of carbon source decline twice the OCV detected foinitial microbial cultures showed differences between the CFU/g soil of SMFC1, SMFC2 and SMFC3, and such an effect could be explained by the microbial community variability at the particle size level in the soil [21]. By inoculating the SRWW, initial OCVs of 29.8, 149.5 and 179.6 mV were immediately generated for SFMC1, SMFC2 and SMFC3, respectively. The difference of potential between anode and cathode based on chemical and biological factors could explain this well [24]. The minimum and maximum OCV reached during the transient stage were 0.9–90.7, 9.6–149.5 and 0.3–179.6 mV for SMFC1, SMFC2 and SMFC3, respectively. The initial and highest peak reached in each SMFC were in accordance with the BOD concentration. In the SMFCs is was also observed that BOD concentration had a major influence on the OCV during the transient stage until day 7; although the OCV ratio is lower in between 5–7 d, afterwards an increasing stage was observed. Such an effect could be explained as the required period for adaptation of the microorganisms to the new conditions in the SMFCs [19,20], an endogenous metabolism as reported Chang *et al.* [15] and the latency stage of the microbial growth related with the bioelectrogenic process. The high variability could also be related with the formation of microchannels in the soil matrix through which the SRWW was flowing during this stage, however, this effect was not studied in depth.

**Figure 2:**
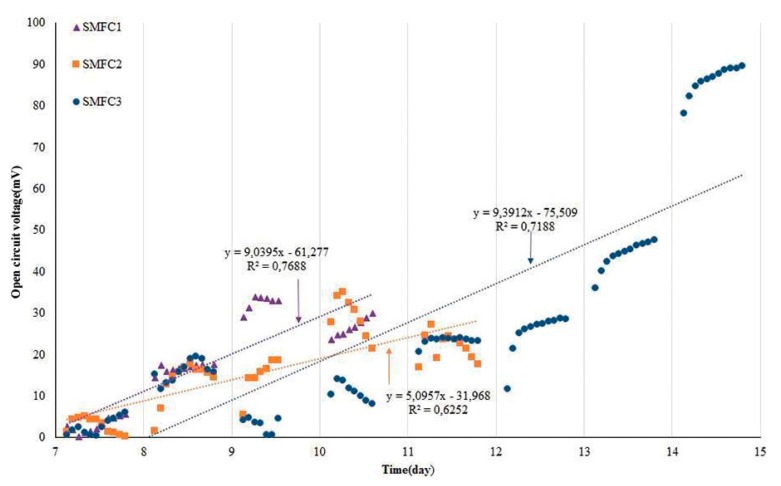
Linear increasing stage of the SMFCs before reaching the steady stage.

In the increasing stage (Phase 2) an independent period of linearity between OCV and time was observed in the SMFCs, with a *R*^2^; of 0.77, 0.63 and 0.72 for SMFC1, SMFC2 and SMFC3, respectively (Figure 2), likely due to enrichment and biofilm formation on the anode electrode. The mean OCV of the minimum and maximum values of this stage increased from 3.04 ± 1.82 mV to 26.63 ± 2.16 mV (SMFC1), 2.79 ± 1.87 mV to 21.86 ± 3.26 mV (SMFC2), 2.65 ± 2.02 mV to 86.14 ± 3.44 mV (SMFC3), and 16.65 ± 9.50 mV to 34.70 ± 2.00 mV (SMFC-control). Furthermore, the ratios of the growing OCV were independent of the amount of microorganisms in the SMFCs (data not shown). In this stage the OCV of the SMFC control, was in a higher ratio than the others, highlighting that the SRWW BOD could influence the OCV output of the other SMFCs in this stage. It also indicates that the microbial community of the Andean soil was the responsible of the electrical response of the SMFCs devices while only consuming the soil nutrients.

According to Peixoto *et al.* 2011 [25] three similar OCVs are needed to consider a steady stage. In previous studies, where the MFCs were entirely exposed into an aqueous environment, 4 weeks [15], 46 d [8] have been required for reaching stable current and potential, respectively. Nevertheless, this study showed that the SMFCs required lesser time than previous studies for reaching a stable OCV output, and it was in dependent on the BOD concentrations. Thereby, SMFC1 reached it first, then SMFC2, however, SMFC3 was still in the increasing stage at the end of the elapsed time, therefore we assumed that the higher BOD concentration tested in SMFC3 caused this effect. Although the control SMFC reached stable OCVs, the measurements during each stable cycle differed each other (data not shown), suggesting that depletion of nutrients could cause this effect.

**Figure 3:**
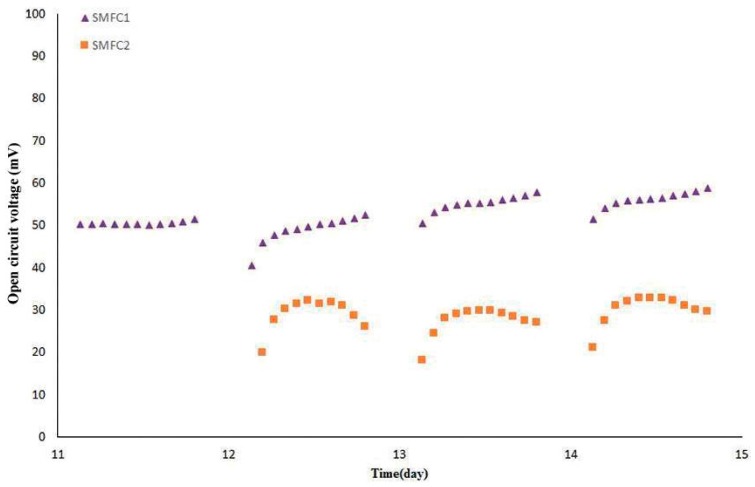
Steady stage and reproducibility of the SMFCs.

Figure 3 depicts the steady stage of the SMFCs inoculated with SRWW. The OCV intervals in this stage were between 40.6–58.8 mV and 18.2–32.9 mV for SMFC1 and SMFC2, respectively. In the meantime, the average OCV in this stage increased 97.82% and 32.20% from the last highest cycle of the increasing stage for SMFC1 and SMFC2, respectively. Results indicated that the OCV of SMFC1 was almost twice that of SMFC2, and required less time to reach the steady stage as well, therefore this suggested that the BOD concentration of the SRWW had the major influence on these terrestrial MFCs, as indicated in a previous report with aqueous MFCs [15]. Although mature biofilm is required for the MFC voltage performance, stability and reproducibility [8], the relative lower OCV in this stage could be caused by over-mature biofilm formation on the anode. Therefore this could reduce the extracellular electron transport to the anode electrode, however, stability was not affected. Moreover, the internal resistance of the SMFCs could be another influencing factor. In the case of the control SMFC the OCV decayed 86.89% from the last cycle in the increasing stage, therefore it was assumed that most of the soil nutrients were depleted. In addition, by contrast to SMFC1 and SMFC2, the control SMFC stabilized at the lowest OCV, likely caused by the fuel cell lack feedstock and the influence of mineral accumulation from the MW, both in the SMFC matrix.

Reproducibility of the SMFCs was observed from day 12 and 13, in four and three cycles for SMFC1 and SMFC2, respectively. Before starting phase 2, after 6 days of SRWW flowing, the CFU/g soil was determined to be 1.2 × 10^11^, 4.0 × 10^12^, 3.0 × 10^11^ for SMFC 1, SMFC2 SMFC3, respectively. Thereby, after six cycles of SRWW flowing through the SMFCs the CFU/g soil was consequently higher than the initial population and likely stable, therefore explaining the linear OCV increment. Figure 2 illustrates the increasing stage of the OCV output with good linearity, due to the fact the number of cells in contact with the anode electrode is proportional to the OCV output [26].

In this study the initial pH of the soil was 5 (*i.e.*, slight acidic). Therefore it was assumed that the microbial population in the SMFCs could be acclimatized to a slightly low pH, although, other studies suggested that the pH should be controlled near neutrality in aqueous-MFCs [27]. In this case, by employing a slight low pH for the adapted microbial population could suggest an advantage, due to the fact acclimatized bacteria could respond earlier, generating the linear increase and steady stages in less time. Nevertheless, it could had some effect on the amount of OCV. Therefore, the major effect of the amount of voltage could be due to nutrient availability.

The results indicated that the SMFCs used in this study required less time to reach stable OCVs than MFCs inoculated with the same Andean soil and organic solid wastes [19,20]. This could suggest the better feasibility of these microorganisms to metabolize simple microbial fuels rather than complex ones. However, the microbial community with organic solid wastes [19,20] showed higher OCVs output than SRWW, likely caused by the MFC design, and the overpopulation of microbes which preferred starch as carbon source, however this dominance could decrease the OCV output. In previous reports, where starch [28], swine [24], rice mill [27] and potato-processing [29] wastewaters have been tested in aqueous-MFCs high performance were achieved. Nevertheless, in this study the linearly increasing and stable OCVs were also reached, but in less time than for aqueous-MFCs. We highlight that the different design and complex matrix made it difficult to compare with other studies (aqueous-MFCs). However, although terrestrial MFCs have been widely tested for producing bioelectricity, through this preliminary investigation we propose their use as biosensors for evaluating BOD.

In the MFC-based biosensor technology parameters like reproducibility, high accuracy and low cost of the devices are important factors to be considered. The MFCs might be suitable devices for in- field monitoring and for this one needs to take into account parameters like temperature, conductivity and pH as Peixoto *et al.* [25] reported. This preliminary study therefore presents terrestrial SMFCs as a useful possibility for environmental monitoring; for instance by using them as a BOD biosensor. Although, lower OCVs output performance were achieved in these MFCs, an increment of the electrical response by a power management system could be applied [18]. In addition, these MFCs depicted positive characteristics such as easy assembly process, lower time for reaching the steady stage and reproducibility for the BOD concentrations of SRWW.

## 4. Conclusions

This study shows that a terrestrial microbial fuel cell-based biosensor could be applied as an alternative setup for monitoring BOD in wastewater. The results indicated that this SMFC had a good and early response for reaching the steady stage and therefore the reproducibility for BOD concentrations lower than 200 ppm of SRWW. It was observed that higher concentrations of BOD required more time to reach the stable phase, suggesting this influencing factor as crucial for consideration in practical applications.
